# Large Evoked Responses and Synchronisation Recorded over the Cerebellum in Human Subjects in Response to Postural Perturbations

**DOI:** 10.1007/s12311-026-02073-4

**Published:** 2026-09-16

**Authors:** James G. Colebatch, Neil P. M. Todd, Daniel Hochstrasser, Peter E. Keller, Sendhil Govender

**Affiliations:** 1 Neuroscience Research Australia, UNSW Sydney, Randwick, Sydney, NSW 2052 Australia; 2 School of Clinical Medicine, Randwick Clinical Campus, UNSW Sydney, Randwick, Sydney, NSW 2052 Australia; 3 MARCS Institute for Brain, Behaviour and Development, Western Sydney University, Westmead, Sydney, NSW 2145 Australia; 4 Center for Music in the Brain, Department of Clinical Medicine, Aarhus University, Aarhus, 8000 Denmark; 5 Institute of Neurological Sciences, Prince of Wales Hospital, Randwick, Sydney, NSW 2031 Australia

**Keywords:** postural reflexes, perturbation, cerebellum, evoked response

## Abstract

In this study we recorded EEG changes over the cerebellum and leg muscle EMG in response to externally applied, stereotyped postural perturbations during standing. Ten healthy adults underwent mechanical perturbations delivered at the shoulders, applied by a torque motor. There was a standing force of 1.17 N and maximum additional force of 18.8 N. Five intermingled conditions were used: four consisted of backwards pulls (75–465 ms duration) and one a “let go” condition (200 ms). EEG was recorded using a cerebellar-extended montage with detailed analyses at the midline Iz electrode. Lower limb EMG (tibialis anterior and soleus) and accelerometry were collected concurrently. Posterior perturbations consistently evoked early postural EMG responses with inhibition of soleus and excitation in tibialis anterior (mean latency 90.2–94.2 ms). Associated with this was a prominent early biphasic cerebellar potential at Iz (P80–N100; mean amplitude approximately 40 µV peak to peak). The properties of this response – its positive onset, location and amplitude – are consistent with Purkinje neuron discharge. RMS averages for EEG activity above 30 Hz revealed patterns localised to the posterior fossa. Responses at Iz consisted of multiple bursts of excitation and inhibition followed by inhibition when the longer perturbations reduced. Single-trial analyses revealed biphasic potentials with short inter-peak intervals (~ 5 ms) synchronised to the excitatory bursts. We conclude that postural perturbations evoke a short latency potential over the cerebellum, likely to represent Purkinje neuron discharge due to climbing fibre input, in turn followed by synchronisation and further facilitation. Our findings support the view that synchronised climbing fibre activity occurs in response to significant sensory events and may facilitate compensatory motor responses.

## Introduction

The Purkinje neurones in the cerebellum are the sole output of the cerebellar cortex and amongst the largest in the nervous system [1]. Eccles et al., [2] showed that Purkinje neurone discharge evoked by a peripheral stimulus and mediated via climbing fibres was associated with a prominent positivity on the surface of the cerebellum, in excess of 200 µV in amplitude (their Fig. 3). Recording evoked responses from the cerebellum has been controversial, partly because peripheral nerve stimuli do not seem to be effective, in contrast to reports of their effects on Purkinje neurones of the anterior lobe of the cerebellum (e.g. Eccles et al., [2]). Others have argued that such recordings are possible, at least for oscillatory activity [3]. While we were unable to evoke a response from stimulating either the superficial radial nerve or the median nerve, we have been able to record reliable evoked responses using vestibular [4] and axial proprioceptive stimuli [5]. Notably the vestibular-evoked cerebellar response (VsCEP) had a biphasic morphology and began with a prominent positivity (P12) followed by a similar amplitude negativity. The mean amplitudes were around 20 µV but were quite variable between subjects. The inter-peak latency was short at 5 ms. Following the response there was a period of inhibition which outlasted the main response and scaled with the evoked response amplitude. Inhibition of tonic Purkinje neuron activity is characteristic of climbing fibre induced responses [6], thus providing support for the assumption that the evoked responses represent complex spikes evoked by climbing fibres (CFR: [7]). Our success with these stimuli probably reflects their projection to the inferior vermis which is close to the surface recording electrodes, more so than is the case for the anterior lobe. Spino-olivary fibres project through the ventral spino-olivary tract to the posterior lobe of the cerebellum, including the vermis [8]. Interestingly, these authors failed to get responses from forelimb nerves whereas they obtained large potentials from the ipsilateral sciatic nerve. These tracts receive projections from muscle afferents (Ib) and cutaneous fibres as well as high threshold afferents [9]. The function of the inferior olive (IO) and its climbing fibre projection remains to be fully clarified. We have shown that even mild truncal stimuli can evoke short latency responses over the cerebellum [5, 10]. In the present experiments we used a much larger perturbation that was a threat to postural stability, expecting it to evoke a more substantial effect. Cerebellar dysfunction is known to be associated with gait ataxia and postural instability (e.g. Morton and Bastian, [11]). Here we report a prominent perturbation-induced discharge, recorded from electrodes overlying the cerebellum in standing humans. This discharge was followed by roughly 10 Hz bursts of excitation and inhibition which showed features of synchronisation. We propose this is a surface manifestation of Purkinje neuron activity and that threats to posture strongly activate the inferior olive and its climbing fibre input to the cerebellum.

## Materials and Methods

### Participants

Ten healthy participants (5 males; mean age 35 ± 15 years) were recruited from staff and students at the Prince of Wales Hospital and Western Sydney University. The same sample size was adequate to detect the evoked responses of interest and we anticipated larger responses given our larger stimulus [5]. All participants provided written informed consent prior to participation. The study was conducted in accordance with the ethical standards of the 1964 Declaration of Helsinki and was approved by the Human Research Ethics Committee of the South Eastern Sydney Local Health District.

### Perturbations and Experimental Procedure

**Fig. 1:**
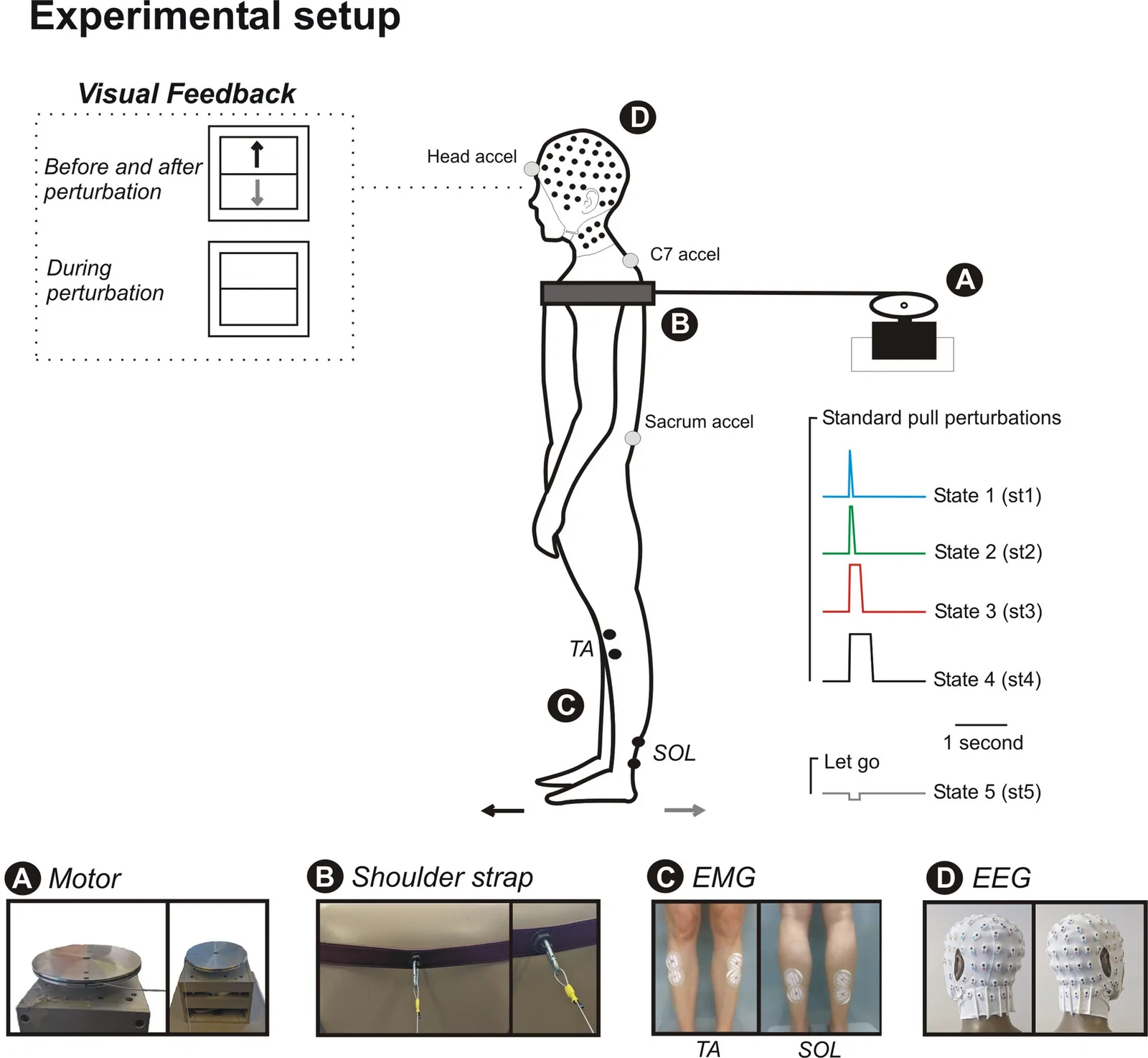
Experimental setup used when recording standard and Let go perturbations. A torque motor (**A**) was used to deliver standard perturbations (states 1–4) in the posterior direction and a Let go (state 5) at the level of the shoulders. Subjects were attached to the motor plate using a belt with hook and wire line (**B**). Subject position was measured using a digital encoder on the motor. Position feedback was fixed to the baseline at the onset of the applied perturbations and for 1.5 s thereafter. EMG (**C**) was recorded from the tibialis anterior (TA) and soleus (SOL) muscles bilaterally. Cephalic recordings (**D**) were made of EEG activity using a customised cap which extended to Iz and the upper neck. Light grey circles indicate linear accelerometer positions at the head, vertebra prominens (C7) and sacrum

These observations were made during a study of the EMG and EEG effects of varying-duration stereotyped perturbations. Four recording blocks were performed, each comprising 82 perturbations with inter-stimulus intervals of 2.5–3.2 s, applied at the level of the shoulders by a torque motor (G12M4H/E Printed Motors. Birmingham, U.K. 0.176 Nm/A; Fig. 1). There was a resting tonic force posteriorly of 1.17 N. There were 5 conditions: for states 1–4 the posteriorly-directed force increased to up to 18.8 N over 15 ms for hold periods of 10, 50, 200 and 400 ms respectively and then fell over 50 ms to the prior tonic level. For state 5 the torque applied went to 0 for 200 ms. The first five perturbations applied served as familiarisation: four posteriorly directed perturbations of increasing duration (st1–4), followed by the anterior “let-go” perturbation (st5). Subsequent trials were pseudorandomised and the sequence repeated across blocks. Participants were instructed to resist the perturbations to maintain their balance and not to take a step. As indicated in the figure, accelerometers were placed over the anterior skull, C7 and the sacrum. Visual feedback from the motor position was provided via an oscilloscope and verbal cues given to ensure the same initial position prior to the start of each perturbation. The visual feedback was fixed at the baseline at the perturbation onset and for the duration of our recording. All trials were performed without footwear, with rest periods between blocks.

### EEG Recordings

EEG was recorded using a 10–10 cerebellar-extended cap (EASYCAP GmbH, Germany) in combination with an ActiveTwo AD-box amplifier and ActiView software (BioSemi, Amsterdam, The Netherlands). Data were referenced to the common average, with detailed analyses conducted for the midline Iz electrode. Recordings were sampled at 4096 Hz, from 0.5 s before stimulus onset to 1.5 s after. The trials were imported into BESA (version 7.1, MEGIS Software GmbH, Germany) and eye blinks removed by modelling an opposite source. The recordings were then exported in CFS format for analysis with Signal (6.02, Cambridge Electronic Devices, Cambridge UK). A high-pass filter at 0.5 Hz and a 50 Hz notch filter were applied to the data prior to detailed analysis. Negative polarities are shown as upward deflections throughout.

### EMG Recordings

Unrectified EMG signals were recorded bilaterally from the soleus and tibialis anterior muscles (TA; Fig.1). Active electrodes were positioned 1–2 cm above the musculotendinous junction for soleus and 1–2 cm lateral to the tibia for TA. Reference electrodes were placed 2 cm below the active electrodes. A ground electrode was placed on the midpoint of the left lower leg. EMG signals were amplified (2500x) and filtered (8 Hz to 1.6 kHz) using AA6 Mk III amplifiers (Medelec Ltd, Old Woking, Surrey UK) then rectified and recorded using Signal software and a Power1401 interface (Cambridge Electronic Design, Cambridge, UK). EMG was averaged offline.

### Data Analysis

#### Averaged Responses

BESA software was used to construct voltage maps based on grand mean EEG data. Amplitudes for the initial positivity were compared across electrode sites in the antero-posterior (AP: Pz, POz, Oz and Iz) and medio-lateral (ML: P9, PO9, I1, Iz, I2, PO10 and P10) directions using repeated-measures ANOVA with electrode as the factor. Unrectified and RMS averages at Iz were used to measure the averaged response peaks in each subject for all perturbations. RMS averages of EEG were made for frequencies of 30 Hz and above as we have previously found this activity is enhanced at electrodes over the cerebellum [5, 12]. This observation is consistent with the tonic discharge rates for Purkinje neuron simple spikes of around 70 Hz [13]. Excitation was defined as a 15% or greater increase and inhibition as a 10% or greater reduction compared to the baseline. Using raster plots, we found some of our subjects showed a single and others multiple discharges at Iz in response to the perturbation and defined two groups based upon the pattern of firing. Acceleration and EEG measures were evaluated using a mixed-model ANOVA with group (defined by single vs. multiple bursts of evoked discharges) as the between-subject factor and recording block as the within-subject factor. For EMG, a mixed-model ANOVA was conducted with group, side (right vs. left EMG) and recording block as factors. Greenhouse-Geisser corrected p values are reported in the text and tables. For averaged responses, mean values reported in the tables reflect the average across the four recording blocks.

### Single Trial Responses

Single-trial data were extracted from all 64 trials per subject using the longest perturbation stimulus (st4). Inspection of these trials showed the presence of small biphasic positive-negative potentials with a short inter-peak interval, resembling the morphology of the evoked P80 potential (see below). These biphasic positive–negative peaks were used to quantify trial-by-trial effects of the perturbation. Raster plots were constructed from single-trials to assess temporal synchrony. Event-related potential (ERP) images were generated using MATLAB (2019b, Mathworks, Natick MA) to visualise response variability. To improve this, data were smoothed using a two-dimensional Gaussian kernel (5 trials × 25 time points, σ = 2). ERP images were then plotted with a symmetric colour scale centred on zero, with positive potentials mapped to red and negative potentials to blue.

## Coherence Testing

Coherence testing between the Iz electrode for st4 and the rectified EMG of the 4 target muscles was performed using MATLAB scripts, based upon *mscohere*. A 1024 point window was used and the data from onset of the perturbation to 1s following, giving a 4 Hz frequency resolution. The results for all 4 leg muscles were combined. Statistical significance was calculated using the mean and standard deviation for coherence results for all frequencies (513 bins) but only the first 25 bins were considered (i.e. up to 100 Hz). Frequencies over 100 Hz would have implied an implausibly short latency difference (< 10 ms). Statistical thresholds of 2.5 and 3 times the SD above the mean were used as significant and highly significant thresholds.

## Results

The shorter standard perturbations (st1 and st2, durations 75 and 115 ms) were perceived to be completed before a voluntary response in the legs was possible. The longer standard perturbations (st3 and st4, hold times 200 and 400 ms) clearly required active resistance. The Let go perturbation (st5) was not always perceived (6 of 10 subjects were unaware) although some subjects felt themselves drifting forwards. None of the subjects required stabilisation for any of the stimuli presented in this study.

The earliest and highest amplitude accelerations occurred at C7 (for states 1 to 4: mean onset 13.1–17.3 ms, mean amplitude 111 mg for state 1 and 174–177 mg for states 2 to 4). Motor shortening varied from means of 3.6 cm (st1) to 7.7 cm (st4), with return to a stable position in the absence of visual feedback over a period of 500 (st1) to 800 ms (st4).

### Grand mean EMG

**Fig. 2:**
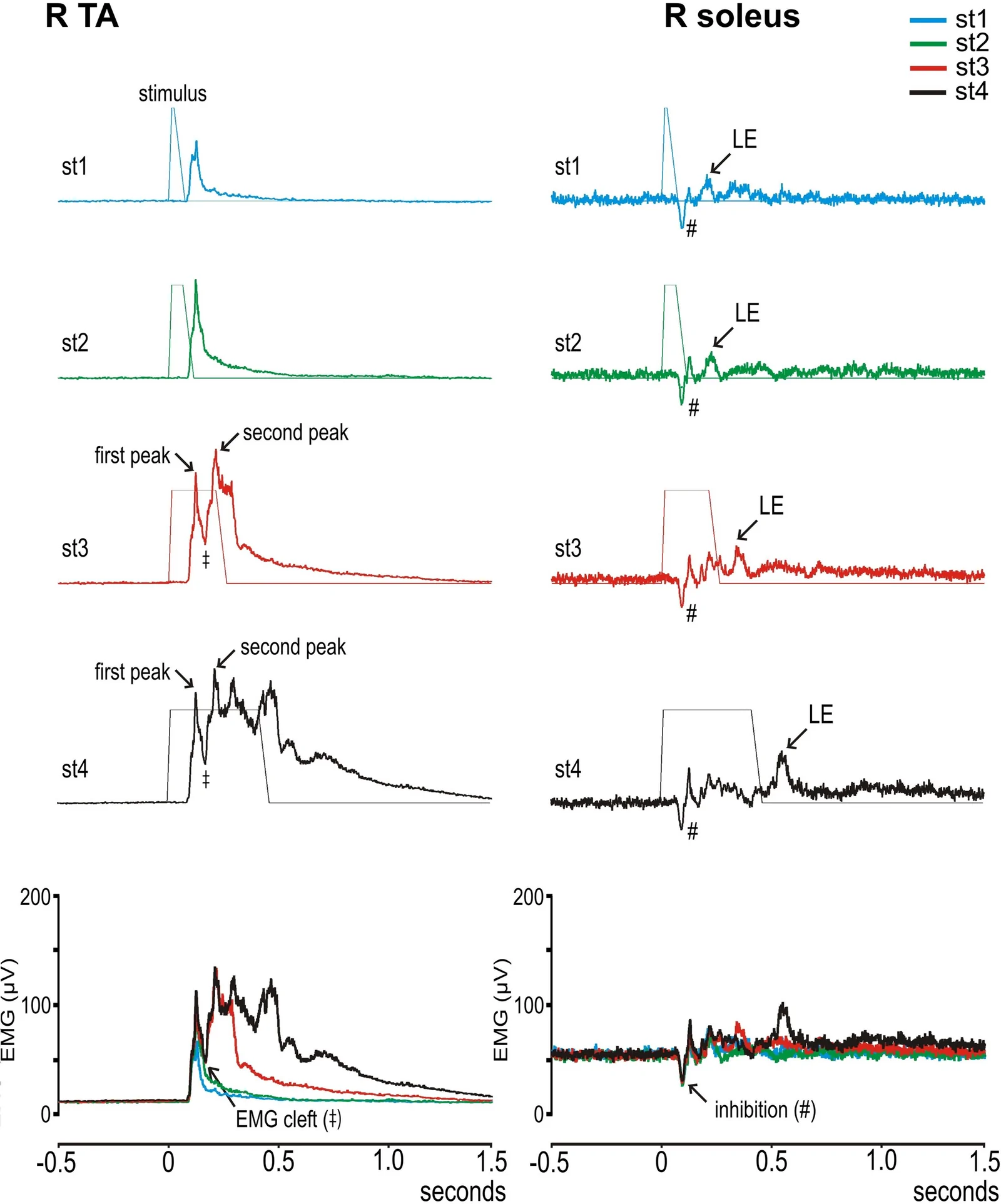
Grand mean EMG profiles (*n* = 10) shown from the right tibialis anterior (TA) and soleus muscle groups for each perturbation state (st1-st4). The EMG response in TA showed a cleft (•) between the early response, present in st1 and st2, separating it from the later responses seen for st3 and st4. Soleus showed an initial inhibition (#) and a late excitation (LE) following the offset of the perturbation

The baseline rearward torque induced a tonic level of EMG in soleus (mean: 51.3–53.1 µV). The earliest response for st1-4 was a period of inhibition in soleus (mean onset: 75.4–77.2 ms, Fig. 2; Table 1). The soleus inhibition appeared to be short-lived but this was probably due to volume conduction of excitation from TA. The onset of excitation in TA was later (mean onset: 90.2–94.2 ms, Table 1) with a stereotyped initial excitation followed by a cleft separating the further bursts of TA EMG activity for states 3 and 4 (mean latency: 170.4 ms (st3) and 170.5 ms (st4)). The peak amplitudes in the early phase of the TA EMG (mean: 140.2 & 157.8 µV) were less than in the second phase (mean: 204.0 & 251.5 µV) (t_(9)_ = 2.3 & 2.8, *p* = 0.046 & 0.021). The EMG activations decayed slowly following cessation of the applied perturbations. For soleus, a short period of late excitation (“LE”) followed the cessation of the perturbation (mean onset latency: 166.4 ms (st1), 193.7 ms (st2), 309.0 ms (st3) and 498.5 ms (st4)). The late excitation appeared to be related to the time of the reduction of applied acceleration, following it by approximately 100 ms.

**Table 1:** Tibialis anterior (TA) and soleus baseline EMG, latencies and amplitudes following pull perturbations (n=10)

	Tibialis anterior parameters			
	st1	st2	st3	st4
Baseline (µV)	13.0 (3.2)	12.7 (3.2)	13.0 (3.4)	14.0 (4.1)
Excitation onset (ms)	92.3 (9.1)	93.6 (8.7)	90.2 (8.4)	94.2 (10.3)
First peak excitation (µV)	98.4 (72.5)	125.5 (75.4)	140.2 (89.5)	157.8 (101.3)
EMG cleft latency (ms)	-	-	170.4 (9.0)	170.5 (7.2)
Second peak excitation (µV)	-	-	204.0 (79.2)	251.5 (87.4)
Second peak end latency (ms)	-	-	310.5 (106.7)	524.9 (17.4)
	Soleus parameters			
	st1	st2	st3	st4
Baseline (µV)	53.1 (19.8)	51.3 (19.8)	52.4 (19.9)	53.1 (19.7)
Inhibition onset (ms)	77.2 (2.9)	75.4 (4.1)	76.8 (5.8)	76.2 (5.1)
Initial peak inhibition (µV)	23.5 (8.3)	25.2 (10.0)	24.2 (9.3)	25.7 (9.6)
Late excitation peak amplitude (µV)	89.7 (32.4)	93.0 (44.2)	99.3 (37.2)	98.9 (47.6)
Late excitation onset latency (ms)	166.4 (21.8)	193.7 (14.8)	309.0 (17.7)	498.5 (15.4)
Late excitation peak latency (ms)	199.9 (26.2)	224.4 (17.1)	352.7 (23.5)	551.2 (13.0)

Mean (*SD*). Values are the mean across the four recordings blocks and averaged across sides. st1-4 = states 1 to 4

### Grand mean EEG

**Fig. 3:**
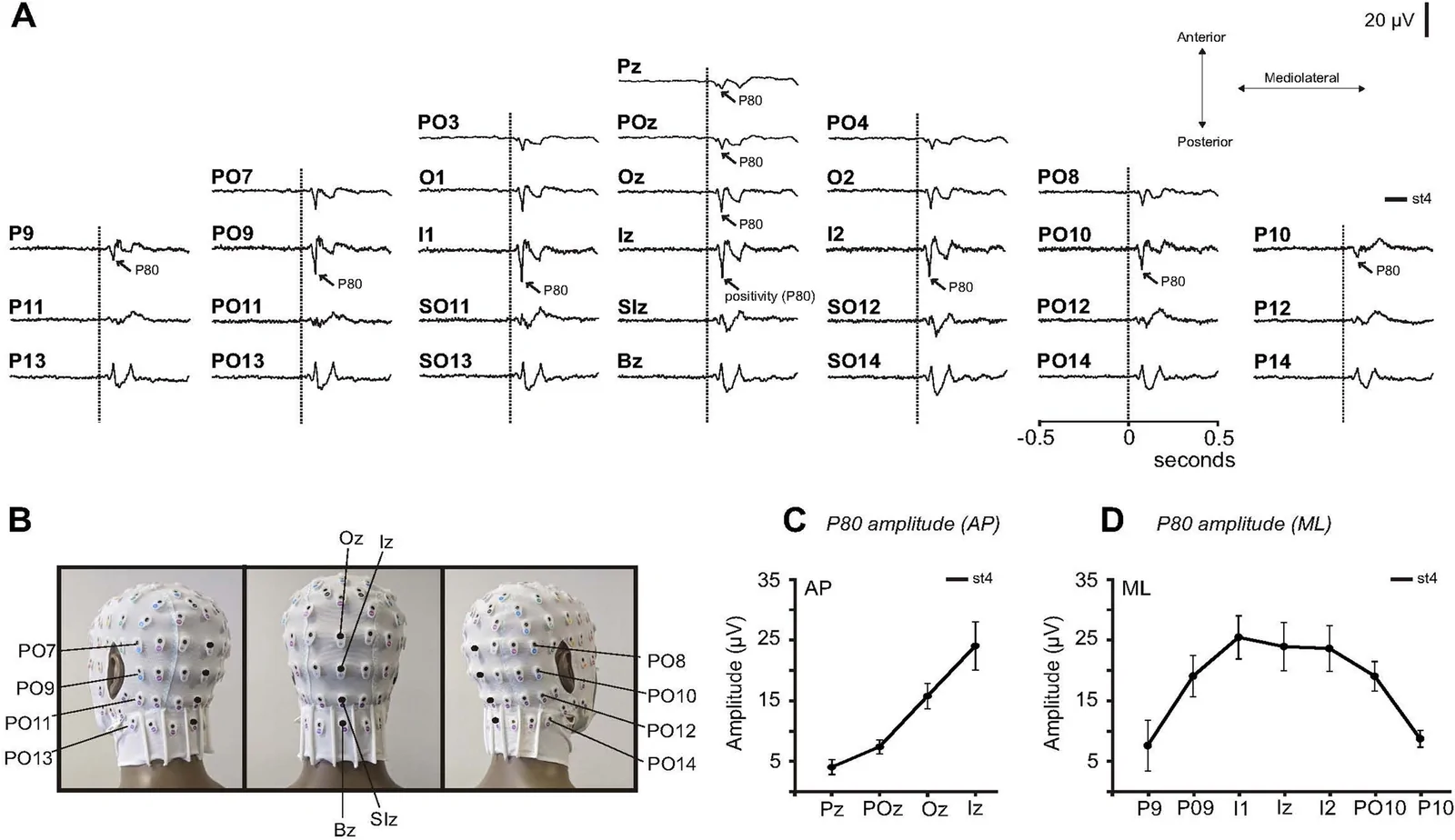
Posterior scalp and cerebellar-extended electrode responses (*n* = 10) to the longest duration perturbation (st4, black traces). (**A**) Averaged waveforms from the parieto-occipital and extended cerebellar sites (Iz, SIz, and Bz rows). The response was maximal around Iz, with reduced amplitudes at adjacent parieto-occipital and occipital electrodes, and polarity inversion at sites below the Iz row. (**B**) Electrode layout of the 10–10 cerebellar-extended cap used for recordings and the electrode positions illustrated in panel A. Mean P80 amplitudes (± SEM) in the anterio-posterior (AP; **C**) and medio-lateral (ML; **D**) directions, showing the maximal response around Iz. Negative potentials are shown as upward deflections

Evoked responses were distributed over the posterior midline and cerebellar-extended electrode sites, with the earliest potentials primarily localized to the posterior fossa region (Fig. 3). A prominent positivity was observed at Iz (P80), which showed polarity inversion at the SIz and Bz rows. This positivity diminished in amplitude both anteriorly (F_(3,27)_ = 24.2, *P* < 0.001; Fig. 3C; ) and laterally relative to the midline electrodes (F_(6,54)_ = 5.5, *P* = 0.007; Fig. 3D).

### Unrectified EEG Averages

**Fig. 4:**
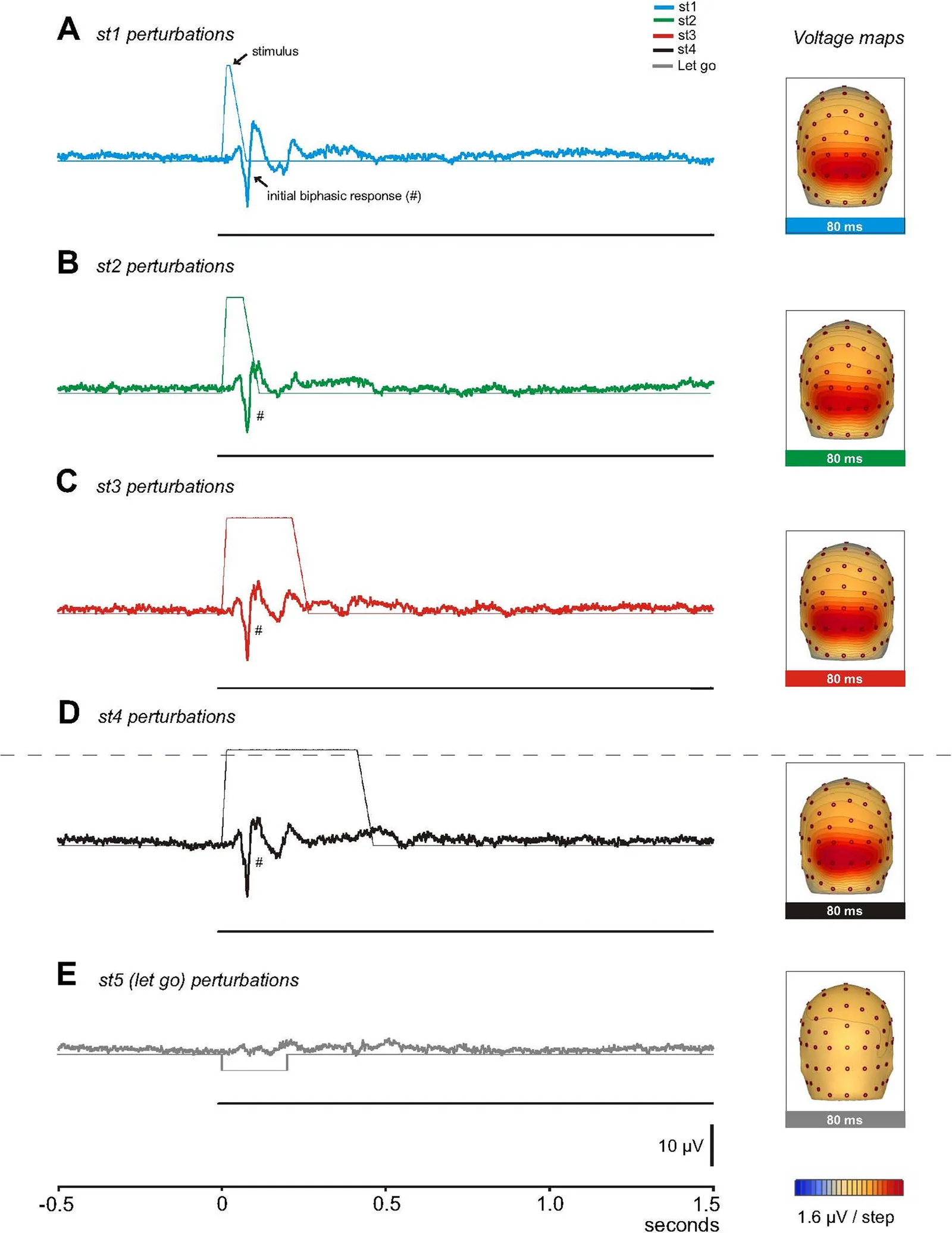
Grand mean EEG responses at the midline Iz electrode (*n* = 10). (**A**–**D**) Responses at Iz to the four posterior perturbations of increasing stimulus duration (st1: blue trace, st2: green trace, st3: red trace and st4: black trace). All posterior perturbations evoked a prominent initial biphasic discharge (#) occurring within ~ 100 ms of perturbation onset. (E) The response to the release (let go) perturbation (st5: grey trace), which did not produce the initial biphasic discharge. For st1-st4 perturbations, voltage maps show the initial positive (red) peak distributed across the posterior fossa. The lines below the traces show when visual feedback was blocked i.e. from the onset of the stimulus to the end of the recording period

Grand mean responses from the midline Iz electrode are shown in Fig. 4 for the four posterior perturbations and the release (“let go”) perturbation. For all posterior perturbations (Fig. 4A–D), a clear early biphasic discharge was observed shortly after stimulus onset, characterized by a sharp positive–negative (P80-N100) potential. This discharge was highly consistent across all posterior perturbations. Voltage maps at the peak of the initial positive deflection (~ 80 ms) revealed a focal distribution centred over the posterior fossa region. The P80 and the following N100 peak amplitudes were unaffected across the 4 recording blocks (*p* = 0.124 & 0.102). In contrast, the let go perturbation (Fig. 4E) did not evoke the initial biphasic response in 7/10 subjects but in 3/10 there were small responses within 10 ms of their latency for state 4.

The P80-N100 potential amplitude was large, with a mean peak-peak amplitude around 40 µV (Table 2), such that it was seen frequently in the single trials of 7 of the 10 subjects (see below). All subjects had P-N responses present for the average for each state – all began with a positivity and 3 had bifid initial positive responses. The mean latency of the first positive peak varied from 62.3 to 87.4 ms (mean 78.4 ms) with an amplitude range of 2.0–39.9 µV (mean 16.2 µV). Individual subject averaged responses showed shorter interpeak intervals than the overall average (14.4 ms, range 8.2–23.5 ms).

**Table 2:** Averaged initial potentials at Iz across posterior perturbation states (*n* = 10)

Averaged potentials at Iz							
	st1		st2	st3	st4	F_(3,27)_	*p*
Peak amplitudes (µV )							
Positivity (P80)		21.3 (11.8)	20.8 (11.0)	21.2 (11.3)	23.6 (14.2)	1.7	0.210
Negativity (N100)		17.8 (9.0)	17.2 (7.8)	18.2 (8.4)	18.0 (10.9)	0.4	0.644
Peak latencies (ms)							
Positivity (P80)		78.6 (5.4)	80.0 (7.1)	79.4 (7.1)	80.4 (8.5)	1.6	0.232
Negativity (N100)		98.0 (9.9)	100.9 (12.3)	101.5 (12.0)	99.0 (10.5)	1.9	0.156

Values are mean (SD) amplitudes and latencies measured from the averaged unrectified trace at the Iz electrode. Statistical outcomes are shown with Greenhouse–Geisser–corrected *p* values. st1–4 = states 1–4

#### EEG RMS Averages

**Fig. 5:**
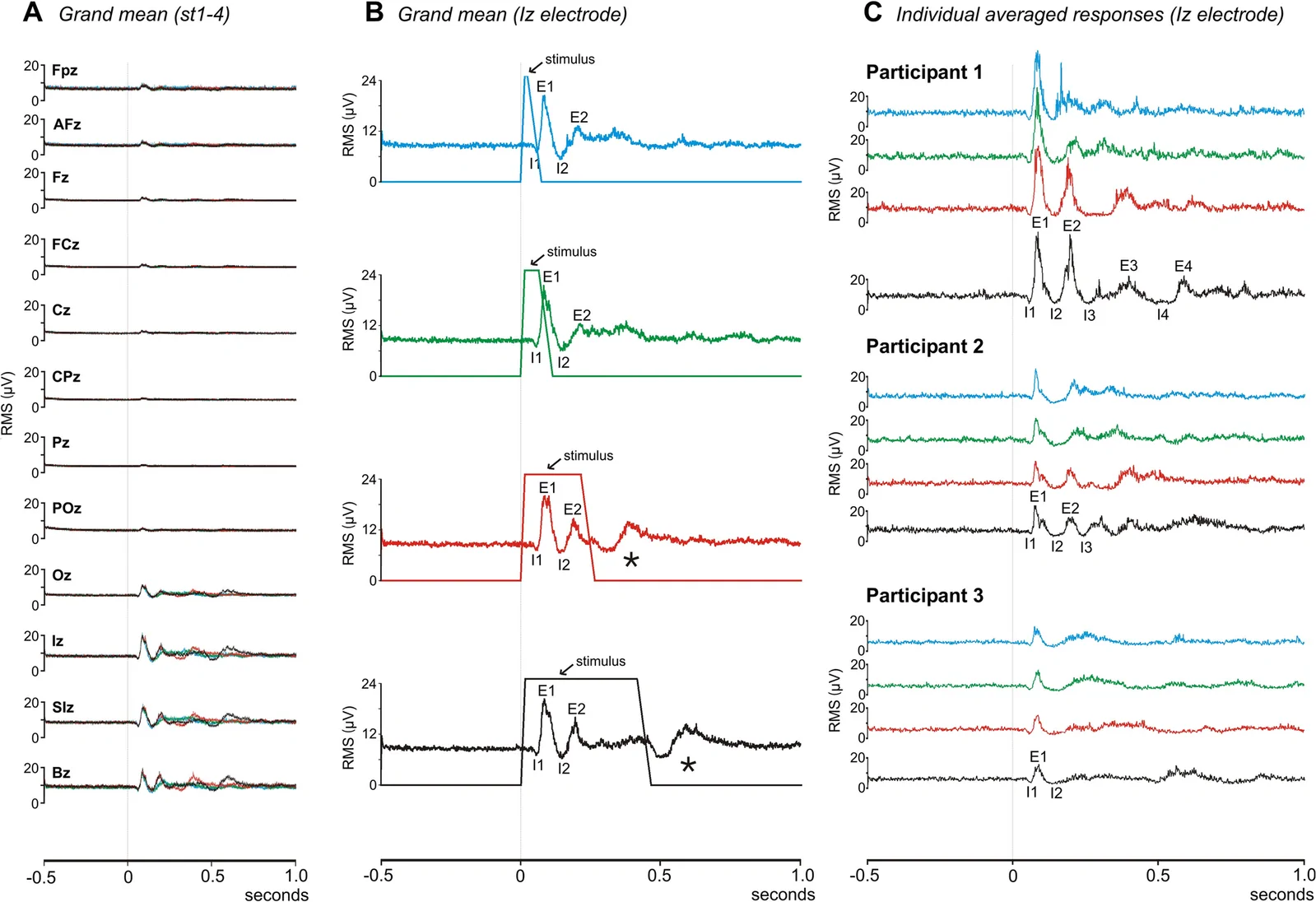
Grand mean RMS averages (*n* = 10, frequencies over 30 Hz) for st1-st4 perturbations (**A**) show clear bursts of activity over the posterior electrodes (Oz to Bz). Grand mean RMS averages at the Iz electrode (**B**) show the initial pattern of activity (I1-E1-I2-E2) across all four perturbations. For the longer durations (st3-st4), an inhibition-excitation was present following the end of the perturbation (*). (**C**) Individual subject RMS averages (st 4) showing varying response patterns at the Iz electrode. RMS averaging revealed multiple excitatory and inhibitory peaks in some individuals (e.g. participant 1 and 2) but not others (participant 3)

Averages of RMS signal power for 30 Hz and above showed a consistent pattern of activity localized to the posterior electrodes (Oz to Bz; Fig. 5A). At the Iz electrode (Fig. 5B), averages revealed an initial inhibition (I1), followed by excitation (E1) and further inhibition and excitation (I2, E2) across all four posterior perturbation states. For the longer perturbations (st3 and st4), there was a delayed period of inhibition, beginning just after the perturbation force was reduced, followed by an excitation. Individual subject responses were more variable, but the E1, I2 and E2 phases were present in nearly all the subjects. The grand mean percentage excitability changes during E1 and E2 were 89% and 47% and for I1 and I2, -22% and − 24%. For state 4, similar to other states, the mean interpeak interval for the E1 and E2 phases of excitation was 127.0 ± 12.7 ms (SD) and between the I1 and I2 inhibitions was 80.0 ± 13.1 ms (Table 3). Averaged responses for individual participants confirmed the overall pattern (Fig. 5C), showing E1/I2/E2 peaks in most, whereas later peaks were variably present.

**Table 3:** RMS baseline levels and peak amplitudes and latencies recorded at Iz following pull perturbations (*n* = 10)

RMS values						
	st1	st2	st3	st4	F_(3,27)_	*p*
Baseline (µV )						
Iz electrode	8.7 (2.3)	8.5 (2.2)	8.6 (2.2)	8.6 (2.2)	3.4	0.081
Peak amplitudes (µV )						
I1 peak	5.1 (1.8)	5.2 (1.6)	5.4 (2.0)	4.9 (2.1)	0.8	0.441
E1 peak	26.0 (11.9)	27.0 (13.6)	28.0 (14.4)	28.1 (13.6)	0.7	0.462
I2 peak	4.9 (2.1)	5.3 (2.3)	4.5 (1.5)	4.3 (1.8)	2.4	0.153
E2 peak	19.4 (9.3)	17.8 (5.6)	20.5 (13.2)	21.7 (13.8)	2.0	0.177
Peak latencies (ms )						
I1 peak	61.7 (5.6)	60.3 (5.6)	61.2 (5.5)	60.4 (4.8)	1.0^	0.368
E1 peak	85.6 (8.2)	87.5 (8.2)	88.9 (9.2)	87.0 (11.2)	1.7	0.216
I2 peak	150.2 (25.8)	157.7 (28.0)	147.8 (15.7)	145.3 (16.4)	2.9^+^	0.112
E2 peak	207.5 (27.8)	220.3 (32.6)	213.2 (27.8)	214.0 (29.6)	1.7^+^	0.224
Prevalence						
I1 peak	7/10	7/10	6/10	7/10	-	
E1 peak	10/10	10/10	10/10	10/10	-	
I2 peak	9/10	10/10	9/10	7/10	-	
E2 peak	8/10	9/10	10/10	8/10	-	

Mean (*SD*). Values are the mean across the four recordings blocks. st1-4 = states 1 to 4. ^ F_(3,18)_ for I1 peak and ^+^F_(3, 24)_ for I2 and E2 peaks

### Individual Responses - Iz

**Fig. 6:**
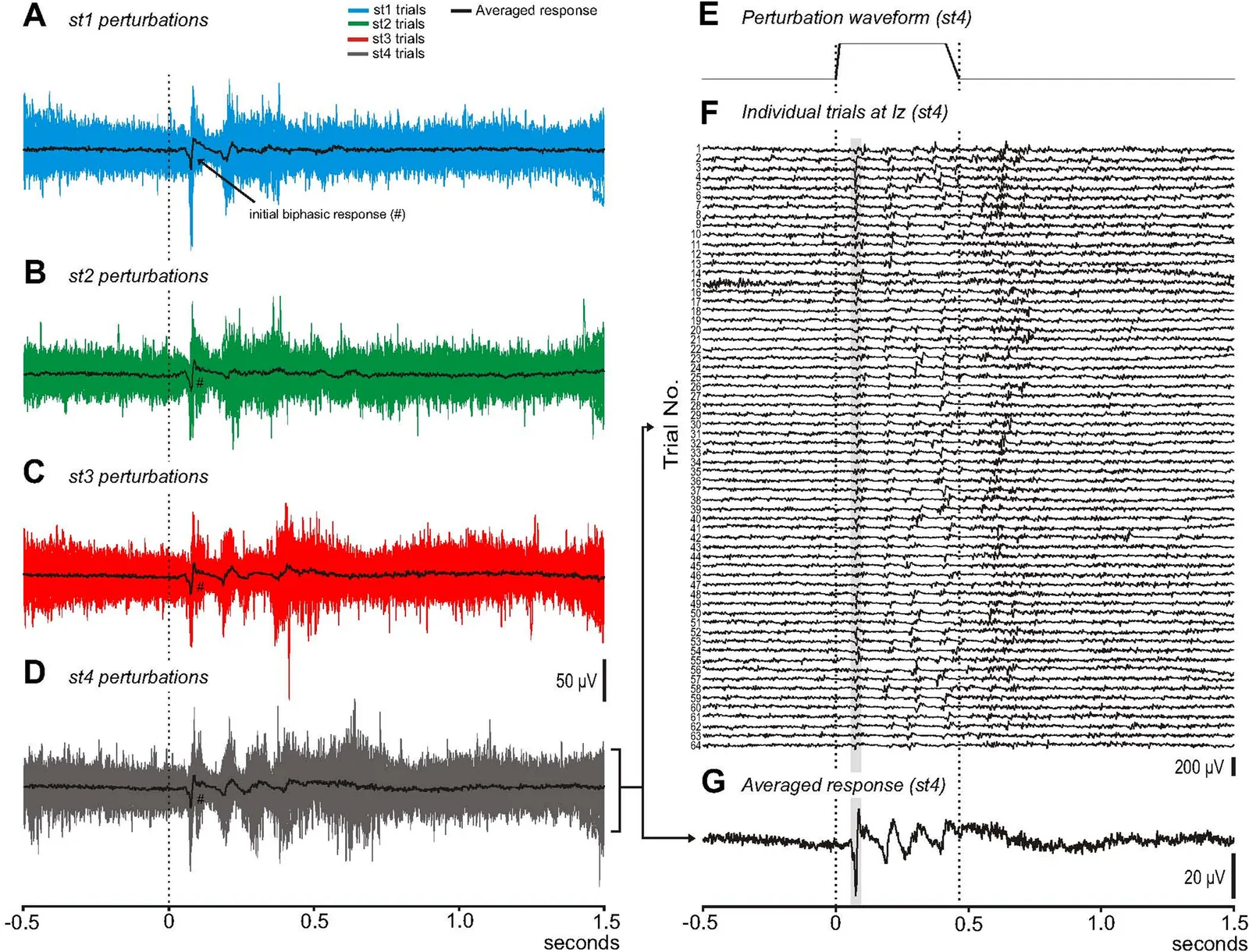
Single subject EEG responses recorded at the Iz electrode. Overlaid individual trials and averaged waveforms are shown for all four posterior perturbations (**A**-**D**; st1-st4), with the initial biphasic response present across all perturbations (left column). As stimulus duration increased, additional later discharges became more pronounced. For the st4 perturbation (right column), the stimulus waveform (**E**), the series of single trial EEG recordings (**F**), and the averaged waveform (**G**) are shown separately. The initial synchronised component of the evoked response is highlighted (grey shaded area) and was present for virtually every trial. Multiple bursts were present after the initial response, clustering in preferred latencies. Vertical dashed lines denote the onset and offset of the perturbation period for the right column. Recordings are shown for participant 1

Figure 6 shows the overlaid individual trials and averaged waveforms from a single participant at the Iz electrode across all four posterior perturbations (Fig. 6A-D). Data is also presented separately for the longest duration (st4) perturbation (Fig. 6E-G). The perturbations elicited an initial highly synchronized, event-related response seen in individual trials (Fig. 6F). The averaged P80 and N100 peaks for this participant were 24.8 and 13.9 µV respectively (Fig. 6G). Beyond this initial synchronous component, a series of similar-appearing rapid positive-negative deflections were present that extended throughout the perturbation period. The potentials exhibited a biphasic waveform, initially positive, with a short inter-peak interval averaging 5.1 ± 0.8 ms across all subjects.

**Fig. 7:**
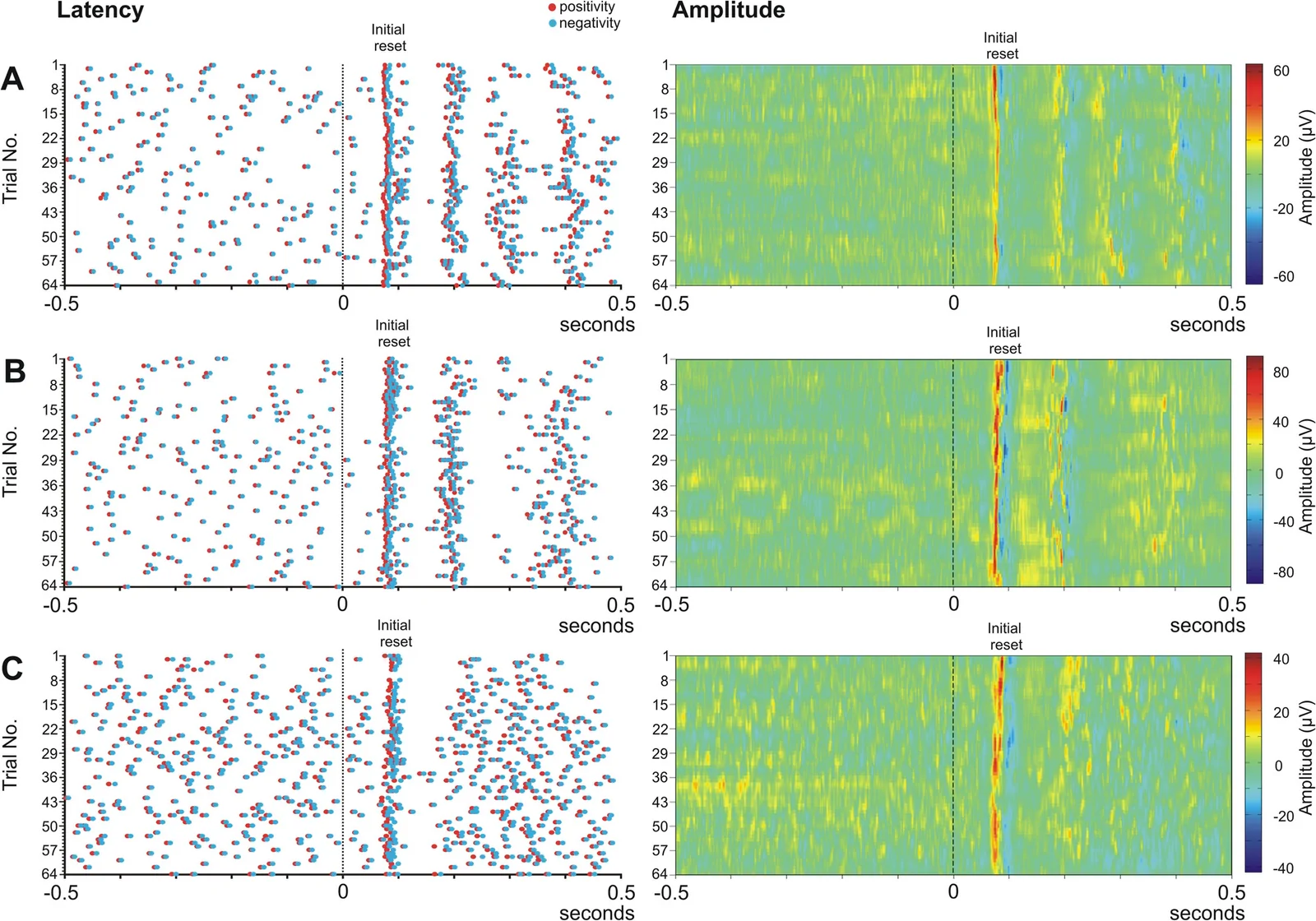
Raster plots of biphasic positive–negative (red-blue) latencies (**A**–**C**) and corresponding ERP amplitude images (right half) are shown for the three participants (top row: participant 1; middle row: participant 2; bottom row: participant 3). Each raster plot displays single-trial latency measurements of biphasic positive-negative potentials. Before the perturbation, responses were unsynchronised across trials, whereas perturbation onset evoked synchronising of potentials, producing early biphasic responses. ERP images (right panel) show the corresponding trial-by-trial amplitude patterns, where the early time-locked positive-negative bands appear within the first 100 ms of perturbation onset. Later potentials exhibited greater variability and reduced synchrony across trials

Individual trial measurements are illustrated in raster plots (Fig. 7, left column) alongside the corresponding ERP images (Fig. 7, right column) for the three representative participants. Prior to stimulus onset, potentials were temporally unsynchronised. The mean inter-peak unitary potential latency ranged from 5.4 to 6.0 ms.

Participant 1 displayed the most consistent trial-to-trial alignment across both early and later potentials, evident as dense vertical groupings (Fig. 7A) and well-defined amplitude bands, persisting beyond the initial biphasic potential. For the initial biphasic potential, mean latencies were 86.8 ± 13.7 ms (positive peak) and 92.8 ± 12.8 ms (negative peak). The second burst occurred at 196.1 ± 11.6 ms and 201.5 ± 11.5 ms, and the third at 291.3 ± 19.9 ms and 297.1 ± 20.1 ms.

In participant 2, later potentials remained moderately synchronised, although with reduced synchrony compared to the earliest biphasic potential (Fig. 7B). Mean latencies were 85.5 ± 11.1 ms and 90.7 ± 10.8 ms for the initial biphasic potential, and 193.9 ± 14.9 ms and 198.8 ± 15.0 ms for the second burst. The mean inter-peak latency was 5.1 ± 1.9 ms (initial potential) and 5.0 ± 1.9 ms (second burst) for participant 2.

In contrast, participant 3 exhibited only a transient synchronisation after the initial reset (85.6 ± 9.5 ms and 92.6 ± 9.5 ms), with dispersed latencies (Fig. 7C) and weaker, more diffuse amplitude patterns. The mean inter-peak latency was 7.1 ± 3.9 ms for the initial biphasic potential. Despite these differences in the later potentials, all three participants showed a consistent stimulus-related discharge for the earliest biphasic potential.

### Single vs. Multiple Synchronised EEG Discharges

Based upon the raster plots, half of the participants (5/10) exhibited a single synchronised burst, whereas the remaining half showed multiple bursts (4/10 with two bursts and 1/10 with three bursts). Those with multiple bursts showed larger peak-to-peak P80-N100 amplitudes (55.6 ± 25.9 µV vs. 27.4 ± 10.2 µV; F_(1,8)_ = 5.5, *p* = 0.047) and earlier N100 peak latencies (92.7 ± 10.5 ms vs. 105.3 ± 10.5 ms; F_(1,8)_ = 5.5, *p* = 0.047) compared to participants with a single burst. Acceleration onset, peak amplitude, and peak latency did not differ between groups at the head (*p* = 0.236–0.867), trunk (*p* = 0.283–0.735) or sacrum (*p* = 0.461–0.632). Similarly, initial peak amplitude and latency in motor displacement were not significantly different between groups (*p* = 0.803 & 0.562). No significant main or interaction effects of group were observed for either TA (*p* = 0.174 to 0.962) or soleus EMG (*p* = 0.361 to 0.742).

### Iz-EMG Coherence

All subjects had significant coherence for the 0–4 Hz bin, probably related to stimulus artefact. Seven of the 10 subjects showed additional significant coherence, 6 for the 12–16 Hz frequency bin and 1 for the 8–12 Hz one. Phase angle analysis indicated that the DC coherence had 0 ms delay, consistent with artifact. With the Iz potential inverted, the 8 and 12 Hz bin phase angles implied latencies between Iz and EMG of a mean of 28.3 ms with 3 subjects having Iz leading and 4 with Iz lagging the EMG responses.

## Discussion

The cerebellum appears to have a role in anticipating the consequences of voluntary motor actions (“feed forward”, e.g. Therrien and Bastian, [14] but our paradigm examines its role in responding to perturbations of posture of varying durations. Maintaining upright stance despite unexpected perturbations is a very important task of the motor system and we have shown here that intense activation occurs over the cerebellum in response to threats to stance. Voluntary reaction times in the legs are of the order of 150 ms [15] by which time a modest perturbation of an average of 0.2 g would have displaced the centre of mass by 2 cm, enough to risk a fall. Responses in the postural muscles, as shown here, start well before this and are likely to be mediated by brainstem pathways. For the limbs, stretch reflexes include the long latency stretch reflex, appearing before a voluntary response to a tap and which in turn appears to be mediated by transcortical pathways (e.g. Marsden et al., [16]). The cerebellum has a role in these reflexes as disease of the cerebellum can enhance these responses [17]. However, it is generally believed that initial postural reflexes are dependent upon the brainstem [18] and our previous findings support this. A short tap over the sternum or C7 vertebra evokes well-formed short latency postural reflexes [5], unlike the response in the limbs to a tap. Postural reflexes, unlike long latency stretch reflexes, do not change with precuing [19]. We have previously demonstrated a spino-bulbar-spinal pathway originating from neck muscle afferents which evokes short latency postural reflexes [20]. This represents one means by which short latency postural responses can occur following a postural perturbation to the upper body. In the present study, the earliest response was inhibition of soleus, which was tonically activated, and occurred around 75 ms after perturbation onset, followed by excitation in TA at around 90 ms. Reciprocal inhibition at the spinal level in response to a single descending volley facilitating TA could underlie this [21]. There appeared to be a nadir of the EMG in TA separating the early and later EMG components, occurring around 180 ms. Colebatch et al., [22], who used manually applied pull-back perturbations, also reported two phases of EMG activity, with a separation at around 200 ms. We have previously proposed that the initial EMG volley is the result of descending activity in reticulospinal fibres [23, 24] as it is too early to result from transcranial reflexes [25]. The EMG cleft in turn is likely to represent the onset of the motor cortically-mediated voluntary EMG response. There was little late EMG for states 1 and 2 which had quite brief perturbations and which were over before the participants could fully react to them. Subjectively, robust voluntary activation was required to maintain upright stance in states 3 and 4 and substantially more late EMG activity was present. 

The perturbation onset was followed by a prominent and short duration positivity (P80), which was large enough to be seen in single trials in some subjects. We did not examine the changes with differing initial perturbation amplitudes, but the response was considerably larger than we obtained using a tap to the upper neck [5]. The biphasic potential always began with a positivity thus the profile was unlike that of a motor unit. The amplitude and interpeak intervals were unlike a visual evoked potential. The consistent phase reversal indicates an origin from a dipole lying between Iz and SIz. We have previously argued that these discharges represent climbing fibre responses (CFRs) evoked from cerebellar Purkinje neurons [7], which are known to have high amplitude surface positive potentials [2]. Armstrong and Harvey, [26] also reported large positive surface potentials arising from Purkinje neurons via olivary projections in response to peripheral stimulation. Purkinje neurones discharge with a mixture of simple spikes, around 50–80 Hz, evoked by mossy fibre inputs, and also complex spikes, evoked by climbing fibres and having low discharge rates [13]. The RMS averages may partly reflect simple spikes, the main form of activity but the E1 peak included the P80 discharge. This was preceded by a brief inhibition (I1) and followed by a clearer period of inhibition, the latter consistent with the effects of climbing fibre-evoked discharge [6]. The P80 peak occurred after the onset of the postural EMG response. It is likely to be analogous to the P54 and P50 potentials shown in response to taps over the sternum and C7, respectively [5, 10], the longer latency being due to the slower rise time of our present perturbation. Our findings have some similarities to those of Andersson and Armstrong [27] who recorded from the lateral vermis in walking cats. The background discharge of Purkinje neurones was low, but a peak occurred following a perturbation caused by the rung on which the cat stood giving way. Purkinje neuron simple spikes have been related to parameters of movement [28, 29], but the role of complex spikes evoked by the climbing fibre pathway from inferior olive to the cerebellum remains to be fully defined. The projection is a unique example in the nervous system in which a single neuron’s activity can excite another as is the case here for the target Purkinje neuron [2]. Theories include an error detection role with either a forward or reverse internal model [9, 30] and a timing role for motor coordination [31]. Direct recordings of climbing fibre activity however have indicated that the activity recorded does not easily fit such proposals. Climbing fibre discharges are slow, typically around 1 spikes/s [13] and rarely exceed 5–10 spikes/s [32]. At the single unit level, climbing fibres demonstrate no increase in response during movement and no definite response to movement error [32, 33], although modulation may be evident at the population level [34, 35]. The inputs to the spino-olivary pathways include high threshold afferents with no input from type Ia muscle spindles [36]. More recently, Sheer and Prsa, [37] have pointed out that, in trained mice, climbing fibre activity was more related to sensory events and reward, rather than motor events. They emphasise the discharge was dependent upon the behavioural significance of the sensory events. The prominent P80-N100 potential recorded here is consistent with such an interpretation, given the potential threat to balance that the perturbation represented. This evoked activity may represent a particularly rapid means of responding to an input, overriding ongoing simple spike activity. In most subjects, the large P80-N100 potential was followed by prominent excitation (bursts) and inhibition (pauses) of activity. These appear to be a common feature following induced cerebellar activity (e.g. Todd et al., [38]). There was a correlation with the size of the P80-N100 potential such that the larger potentials were followed by more excitation/inhibition cycles. It appeared that subjects with the bursting discharges had a lower threshold for the stimulus. Whether this means a more effective response to the perturbation is not known but we found no correlation with the EMG responses. The P80-N100 potential itself was preceded by a period of inhibition. The output of the Purkinje neuron is inhibitory [39] and cerebellar stimulation inhibits the motor cortex [40]. Stimulation of the superior cerebellar peduncle, which carries the axons of many of the cerebellar nuclear neurons, evokes excitation in some target neurons but inhibition more commonly so despite selective excitation, the net effect may appear to be inhibitory [41]. In addition, cerebellar nuclear neurons show intense rebound excitation following inhibition, proportional to the level of preceding inhibition [42]. The effect therefore of a short inhibition of the cerebellar nuclear neurons may actually be to induce a subsequent intense excitation in their target group of neurons in the motor and premotor cortices. Inferior olivary neurons typically show multiple spikes following afferent input [43]. Their activity can be reset in vitro using electrical stimuli [44], a property of their connexin36 dendro-dendritic gap junctions which facilitates synchronisation [44, 45]. At a population level, there can be a synchronised 10 Hz rhythm, even though individual IO neurons and their Purkinje targets, only fire around 1 Hz [46]. A corollary of this is that the synchronised amplitude would be only 1/10 of the whole population output. The pattern of discharges following the large amplitude P80-N100 potential is consistent with a similar mechanism of synchronisation and the interpeak peak excitatory and inhibitory discharge intervals approximated 100 ms (10 Hz). Synchronisation of climbing fibre input may be a way of amplifying their effects and facilitating motor output [45]. Purkinje neurons are not only large but also a single climbing fibre appears to contact around 6 or more Purkinje neurons [47, 48], making this the unit of discharge for climbing fibre inputs and amplifying any consequent surface potential. We were able to identify small biphasic, positive-onset potentials which may represent small groups of Purkinje neurons and these too demonstrated synchronisation and facilitation by our perturbation. The morphology and inter-peak interval of these small potentials were similar to that of the evoked response to vestibular stimulation (P12-N17, [4]). 

Cerebellar abnormalities are associated with impaired postural response magnitudes but do not cause abnormal response latencies in leg muscles [49]. Analogous to this, the neural pathway for the blink reflex lies within the brainstem [50] but cerebellar control is sufficient to evoke conditioned responses [51]. A cerebellar contribution via the brainstem is not excluded for the later parts of the EMG response and we showed coherence between cerebellar activity and the leg muscle EMG. Cerebellar activity can sometimes precede that of the motor cortex [52]. The main output from the fastigial nucleus is to the reticular formation and vestibular nuclei [53] but projections also occur to prefrontal cortex [54]. It may thus be that the significance of this potential is also for its role in upstream signalling, indicating an event potentially requiring voluntary intervention. Late averaged EEG peaks were also present at the end of the perturbation, corresponding to the post-perturbation excitation (Fig. 5), but may have arisen from a different population of Purkinje neurons from the earlier ones. As noted above, the evoked responses could be regarded as an overriding or resetting of Purkinje activity, giving priority to the compensatory responses required from both brainstem and cortex. The persisting activity could indicate the continuing need for attention to and correction of the disturbance. This excitation was dependent upon the peripheral input, as evidenced by the inhibition that occurred when the perturbation was tapering. Our findings are consistent with Gibson et al.,’s [32] proposal that “climbing fibres signal externally imposed disturbances to particular regions of the body when the animal is not actively moving”. We are the first to show evidence of intense discharge of presumed vermal Purkinje neurons occurring in response to perturbations in standing human subjects, confirming an important role for the cerebellum under these circumstances. The initial excitatory burst was followed by evidence of further facilitation and synchronisation of climbing fibre discharge. This occurred when the postural disturbance required a rapid and sustained response to maintain stance and appears to reflect properties of the inferior olive and its climbing fibre projection. The compensatory response to sudden and prolonged postural disturbances is likely to require both brainstem and cortical contributions and this is likely to be mediated in part through cerebellar activity.

## Data Availability

Anonymised data will be made available in response to reasonable requests.

## Declarations

Approval was obtained from the Human Research Ethics committee of the South Eastern Sydney Local Health District. The procedures used in this study adhere to the tenets of the Declaration of Helsinki. All participants gave their informed consent to participate.
